# Animalculæ of Typhoid Fever

**Published:** 1864

**Authors:** 


Animalculæ of Typhoid Fever.—Professor Tigri, of Sienna, in Italy, has addressed a paper to the Academy of Sciences of Paris, wherein he declares that he has again found on the bodies of persons who had died of typhoid fever infusoria of the genus Bacterium.

